# Tuberculous Infection of Thyroid Gland: A Case Report

**DOI:** 10.1155/2009/416231

**Published:** 2010-02-04

**Authors:** Prince Cheriyan Modayil, Anna Leslie, Antony Jacob

**Affiliations:** ^1^Department of Otorhinolaryngology, University Hospital Lewisham, Lewisham, SE13 6LH, London, UK; ^2^Department of Radiology, University Hospital Lewisham, Lewisham, SE13 6LH, London, UK

## Abstract

*Introduction*. Tuberculosis is one of the leading causes of morbidity and mortality and almost one-third of the world is infected with this disease. Tuberculosis has been reported in many parts of the human body. But thyroid gland involvement is extremely rare and its true incidence is unknown. *Case Presentation*. We present the case of a 26-year-old woman who presented with a thyroid cyst which turned out to be a primary mycobacterium tuberculosis infection. *Conclusion*. The correct diagnosis of thyroid tuberculosis is important because of the availability of medical treatment and the limited role of surgery.

## 1. Introduction

Tuberculosis of thyroid gland is extremely uncommon. Ultrasound guided fine needle aspiration cytology (FNAC) of the lesion forms an important mode of investigation in its diagnosis. Recognition of this condition can avoid surgery, as the main mode of treatment is medical.

## 2. Case Presentation

A 26-year-old Asian woman currently residing in the UK presented to the ENT outpatients department with a lump on the right side of her lower neck. She noticed the swelling six weeks prior to presentation and it had been gradually increasing in size. She had no systemic symptoms and had a good apetite and her weight had been stable. Her past medical history was insignificant apart from a diagnosis of polycystic ovaries. 

On examination there was a nontender firm to hard lump in the right thyroid lobe, measuring 4 × 4 cms. The rest of the ENT examination including flexible nasolaryngoscopy was normal. There was no other lymphadenopathy and the respiratory, cardiovascular, and abdominal examination was unremarkable.

**Figure 1:**
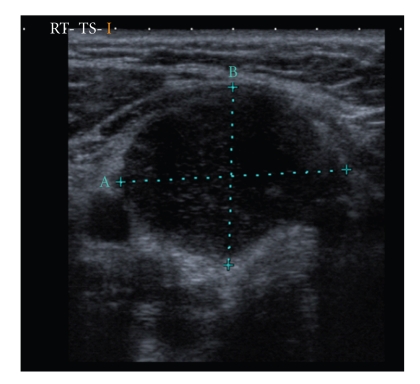
Cystic mass with internal echoes lying anterior to thyroid.

**Figure 2:**
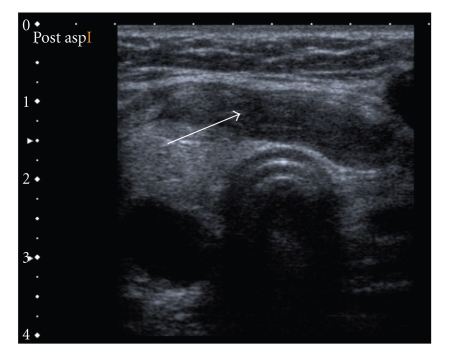
Transverse view of neck showing thick walled cystic mass post aspiration.

**Figure 3:**
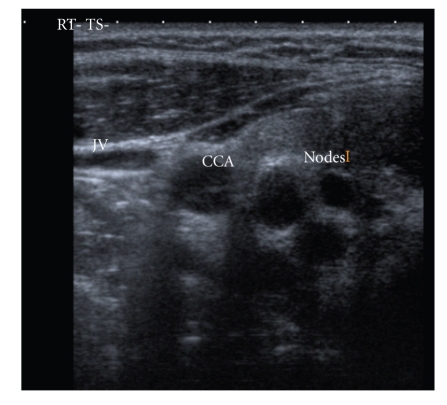
Right lateral neck showing abnormal nodes.

The routine laboratory test results and thyroid function tests were normal except for a raised ESR of 40 mm/hr. Ultrasound examination of the neck revealed a 35 × 18 mm cystic mass in the lower pole of the right thyroid lobe with internal echoes (Figures 1 and 2). An ultrasound guided FNA of the above mass revealed 10 cc of frank pus which raised the clinical suspicion of TB, and subsequent culture was positive for mycobacterium tuberculosis. Ultrasonogram also revealed some abnormal lymph nodes in the right level 2 area of neck (Figure 3). Her mantoux test was 34 mm. Chest X-ray was normal. This patient was referred to the chest physicians and was started on standard quadruple therapy.

### 2.1. Followup

She was seen recently approximately 12 months post treatment and has responded well to treatment and her neck swelling has disappeared. She is euthyroid. A repeat ultrasound showed complete resolution of the neck mass and lymph nodes.

### 2.2. Discussion

Although tuberculosis has been reported in many parts of the human body, thyroid involvement is extremely rare and its true incidence is unknown. The rarity of this disease is attributed to various factors including bactericidal property of colloid material and high thyroid blood flow [1].

Tuberculosis of the thyroid gland may be primary or occur in association with tuberculous infection of other organs [2]. It can present as multiple thyroid lesions associated with military tuberculosis, solitary caseating thyroid nodule, cold abscess, chronic fibrosing type, and acute abscess [2].

The clinical presentation of thyroid tuberculosis can range from being totally asymptomatic to solitary, multinodular goitre with concomitant pulmonary tuberculosis, meningitis, dysphagia, or pyrexia of unknown origin. It can sometimes mimic thyroiditis and cancer. Most of the patients are euthyroid. A high ESR and a positive mantoux test may suggest tuberculosis [2].

Ultrasound guided fine needle aspiration cytology (FNAC) is a useful diagnostic method in thyroid tuberculosis. It is our practice to perform FNA on almost all neck lumps and it is the aspirate of frank pus in a high-risk patient that raised the suspicion of tuberculosis in our patient. 

The diagnosis has to be substantiated by histopathologic findings of caseating granuloma and/or identification of AFB on culture. Other investigation modalities include chest X-ray, sputum analysis, PCR, and cultures with ^14^C-labelled compounds.

Treatment of thyroid tuberculosis does not differ from other forms of this disease. 

Antitubercular drugs remain the main stay of treatment. The concomitant use of two or three effective drugs has to be prolonged for at least six months with appropriate followup. Surgery may be indicated in acute abscesses to avoid total destruction of the thyroid gland.

If left untreated, it can cause complete destruction of the thyroid gland and result in hypothyroidism. The thyroid hormone levels should be monitored before, during, and after treatment.

## 3. Conclusion

Extrapulmonary tuberculosis presents a diagnostic dilemma because of the relative infrequency of occurrence at certain sites including thyroid gland. A possible differential diagnosis of thyroid tuberculosis should be borne in mind while treating patients with thyroid lumps. Ultrasound guided fine needle aspiration cytology (FNAC) is a useful diagnostic method in the diagnostic work up of all neck lumps and as demonstrated here helped to avoid unnecessary surgery.
